# A multiple sclerosis disease progression measure based on cumulative disability

**DOI:** 10.1177/1352458520988632

**Published:** 2021-01-25

**Authors:** Ali Manouchehrinia, Elaine Kingwell, Feng Zhu, Helen Tremlett, Jan Hillert, Ryan Ramanujam

**Affiliations:** Department of Clinical Neuroscience, Karolinska Institutet, Karolinska University Hospital Solna, Stockholm, Sweden; Faculty of Medicine (Neurology), UBC Hospital, and Djavad Mowafaghian Centre for Brain Health, University of British Columbia, Vancouver, BC, Canada; Faculty of Medicine (Neurology), UBC Hospital, and Djavad Mowafaghian Centre for Brain Health, University of British Columbia, Vancouver, BC, Canada; Faculty of Medicine (Neurology), UBC Hospital, and Djavad Mowafaghian Centre for Brain Health, University of British Columbia, Vancouver, BC, Canada; Department of Clinical Neuroscience, Karolinska Institutet, Karolinska University Hospital Solna, Stockholm, Sweden; Department of Clinical Neuroscience, Karolinska Institutet, Karolinska University Hospital Solna, Stockholm, Sweden/Department of Mathematics, The Royal Institute of Technology, Stockholm, Sweden

**Keywords:** Multiple sclerosis, progression measure, outcome, disability

## Abstract

**Background:**

Existing severity measurements in multiple sclerosis (MS) are often cross-sectional, making longitudinal comparisons of disease course between individuals difficult.

**Objective:**

The objective of this study is to create a severity metric that can reliably summarize a patient’s disease course.

**Methods:**

We developed the nARMSS – normalized ARMSS (age-related MS severity score) over follow-up, using the deviation of individual ARMSS scores from the expected value and integrated over the corresponding time period. The nARMSS scales from −5 to +5; a positive value indicates a more severe disease course for a patient when compared to other patients with similar disease timings.

**Results:**

Using Swedish MS registry data, the nARMSS was tested using data at 2 and 4 years of follow-up to predict the most severe quartile during the subsequent period up to 10 years total follow-up. The metric used was area under the curve of the receiver operating characteristic (AUC-ROC). This resulted in measurements of 0.929 and 0.941. In an external Canadian validation cohort, the equivalent AUC-ROCs were 0.901 and 0.908.

**Conclusion:**

The nARMSS provides a reliable, generalizable and easily measurable metric which makes longitudinal comparison of disease course between individuals feasible.

## Introduction

Multiple sclerosis (MS) is a chronic, neurodegenerative disorder of the central nervous system which requires lifelong care. As MS has a heterogeneous disease course, it is important to determine a patient’s current and potential future severity in order to understand if interventions affect the disease course and to mitigate progression of disease. The most commonly used disability measure in both MS clinical practice and clinical trials is the expanded disability status scale (EDSS). The EDSS is an ordinal scale ranging from 0 (no neurological deficits) to 10 (death due to MS) and includes an assessment of eight functional systems by a neurologist during a clinical examination.^1^ Due to limitations of the cross-sectional nature of the EDSS score, the MS severity score (MSSS)^2^ and age-related MS severity score (ARMSS)^3^ were created which enable a patient’s EDSS to be ranked by years since disease onset or age, respectively. Compared to the MSSS score, the ARMSS may be more versatile as, instead of the date of MS symptom (which is often missing or imprecise), an individual’s age is used. This may allow for inclusion of more patients within a given cohort. Also, use of the ARMSS score could eliminate any bias or imprecision introduced by the need for data on disease duration. However, there is still a need for more accurate disease severity and progression measurements that capture a complete overview of a patient’s disease, regardless of when they are measured.

There has been a notable lack of a severity score to directly compare patient’s overall disease course without undue sensitivity to follow-up time. The combinatorial weight-adjusted disability (COMBIwise) score was one notable effort to create a measure to predict future disability based on several criteria measured early in a patient’s follow-up.^4^ Although it made some progress towards a predictive score, some limitations were present such as (1) the requirement of precise information for the calculation, thereby reducing patient inclusion and (2) its comparatively low accuracy at predicting future disability.^5^ In addition, an early effort to use serial EDSS measurements to construct a single metric based on area under the curve as an outcome for clinical trials did not attain widespread use.^6^

Hence, we sought to use ARMSS to build a more comprehensive metric that would give a cross-sectional view of disease. The aim of this study was to investigate the performance of this new measure, normalized ARMSS (nARMSS), which we developed in order to create an instant overview of a patient’s course as well as to provide a potentially enhanced ability to predict future disability. This score can be calculated solely from EDSS/ARMSS early in as well as throughout follow-up and shows strong correlation between early and future disability.

## Materials and methods

This study included individuals with MS from two large clinical cohorts. The first, the Swedish MS registry (Swedish cohort) is a National registry of patients diagnosed with MS based on the McDonald criteria.^7^ The registry is voluntary, and the vast majority of neurologists in Sweden electively participate, resulting in the inclusion of nearly 85% of MS patients in Sweden.^8^

To be included in the study, individuals were required to have complete data on sex, date of birth, date of disease onset (first manifestation of MS) as well as >1 EDSS score (and the date of EDSS capture). These data were used to calculate the ARMSS against a published reference matrix of global values,^3^ using the R package ms.sev version 1.0.4.

In this study, 20,025 individuals in the Swedish cohort with 121,616 clinical visits which included an EDSS measurement were eligible, of which 14,160 individuals were included based on the above criteria.

The second cohort for validation was from British Columbia, Canada (Canadian cohort) and has been previously described.^3,9,10^ This cohort comprised 5989 eligible individuals.

### Calculation of the nARMSS

The nARMSS was constructed based on serial EDSS scores for a patient and required at least two scores for calculation. Follow-up was defined as the time from the first recorded EDSS to the most recently recorded EDSS for all patients. First, an ‘ARMSS integral’ was calculated as the difference in the integrated area under the ARMSS scores from the expected median ARMSS of 5. The ARMSS integral can therefore be thought of as the area under the curve for all ARMSS measurements during follow-up in relation to the median value of 5. Positive values indicate an accumulation of disability greater than average for the patient’s age(s) of disease. The equation for ARMSS integral is

$$\int_{age_{1}}^{age_{n}}ARMSS_{age}-\left[(age_{n}-age_{1)}\times 5\right]$$

where n is the total number of ARMSS scores, $age_{1}$ is the age at first EDSS measurement, $age_{n}$ is the age at last EDSS measurement and $ARMSS_{age}$ is the ARMSS score at a given age.

**Figure 1.**
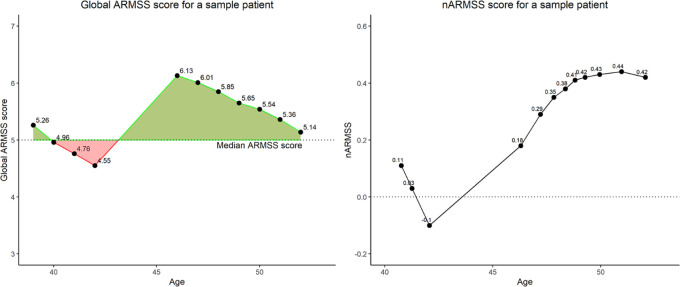
Example of the construction of nARMSS using serial ARMSS scores for a given patient. The ARMSS integral (left) is determined by the areas in green less the areas in red. The nARMSS can be calculated (right) and is shown longitudinally.

The ARMSS integral can be considered a relative measure for the total disability experienced by a patient over follow-up years. In order to compare patients when ages and follow-up time are different, the nARMSS is the ARMSS integral which has been normalized using follow-up time. Figure 1 illustrates a sample calculation based on a single patient’s EDSS and ARMSS scores.

The formula for the nARMSS is

$$\frac{1}{\left(age_{n}-age_{1}\right)}\left(\begin{array}{l} \int_{age_{1}}^{age_{n}}ARMSS_{age}\\ -\left[(age_{n}-age_{1)}\times 5\right] \end{array}\right)$$

As such, the score varies between +5 and −5, since the ARMSS is scaled between 0 and 10 and the ARMSS integral is determined based on the deviation from 5. The nARMSS is therefore the normalized ARMSS over follow-up relative to median, and each increase in one unit gives the average increase in the ARMSS (and thus decile) from the average patient with identical disease timings.

An R shiny app for calculating nARMSS directly from serial EDSS scores for an individual can be found online at https://aliman.shinyapps.io/nARMSS/.

We also determined the ranges of ARMSS and nARMSS for all individuals in the Swedish cohort, defined as the difference in maximum and minimum values over follow-up. This was calculated to determine if any reduction in the range of nARMSS was present over ARMSS. These were then compared to determine the compression of the range in the nARMSS relative to ARMSS.

### nARMSS association to future disability

We sought to determine if nARMSS early in follow-up could predict nARMSS for a patient’s next years until 10 years of follow-up, without overlapping data. The following procedures were therefore conducted in both the Swedish and Canadian cohorts.

nARMSS at 2 and 4 years were then used to predict nARMSS during the next 8 and 6 years of follow-up, respectively. The outcomes were recoded as binary where the most severe quartile of patients was coded as 1, and patients with scores in the remaining quartiles were coded as 0. To maximize the number of patients included, at 2 and 4 years of follow-up, the closest chronological EDSS was used (ranging from 0.5 to 3 and 2 to 5 years of follow-up, respectively). We used area under the curve (AUC) of the receiver operating characteristic (ROC), a metric constructing using the curve of sensitivity and specificity, to determine how well the nARMSS was associated with future disability. This procedure was repeated for nARMSS at 8 years to predict the following 7 years of follow-up, that is, 15 total years, as a final check of the reliability of this method.

Similar methods were conducted using both the MSSS and EDSS values at 2 and 4 years to predict the most severe nARMSS quartile over the next years to 10 years total follow-up, resulting in four separate AUC-ROCs per cohort. This was repeated using the average EDSS for all visits up to the timepoint used in the 2 and 4 year cutoffs to predict identical Q4 nARMSS binary outcomes. The average MSSS was also tested in an identical manner to average EDSS, in order to determine the Q4 nARMSS predictive ability.

Finally, the same approach was applied using secondary progressive MS (SPMS) as the outcome, coded as 1 for SPMS and 0 for those who had not converted to SPMS, as determined retrospectively by a neurologist. This included all previous tests using both the 2 and 4 year timepoints, with the outcome being SPMS status after 10 years of follow-up. Individuals who were SP at the 2 or 4 year timepoint were removed from the analysis.

### nARMSS comparison to other MS outcomes

The various quartiles of nARMSS were compared to other measurements that are often used as severity outcomes in MS studies. The mean values of nARMSS, EDSS, first SDMT (Symbol Digit Modalities Test) ever completed at any point during follow-up time and first ever MSIS-29 (multiple sclerosis impact scale-29) physical and psychological scores were compared for patients in nARMSS quartiles, defined as the last nARMSS value. These were repeated for quartiles of EDSS and MSSS, defined as the average values over entire the follow-up. The SDMT provides a standardized measurement of cognitive ability.^11^ Since a learning effect from repeated testing has been noted, only the first measurement was used. The MSIS-29 is a self-assessment consisting of 29 questions covering physical, psychological and well-being.^12^ Similarly, the first scores for physical and psychological symptoms were measured separately. The SDMT and MSIS-29 were only available for the Swedish cohort, as these have become part of the routine clinical assessment in Sweden since the introduction of the second generation of DMTs in 2006.^13^

### Data availability

Data from the Swedish MS registry used in this article can requested from the Karolinska Institutet. This requires both a data transfer agreement and required ethical permission facilitated between Karolinska Institutet and the institution requesting access to the data in accordance with the data protection legislation governing Europe, GDPR (General Data Protection Regulation). Researchers who are interested in obtaining data access should contact the corresponding author.

## Results

Overall, we included 14,160 patients from the Swedish cohort and 5989 patients from the Canadian cohort. Sub-analyses used reduced sets of patients according to available data as indicated. Characteristics of the study population are presented in Table 1.

**Table 1.** Characteristics of the Swedish and Canadian cohorts.

	Swedish cohort	Canadian cohort
*n*	14,160	5989
Age at MS onset (mean (SD))	33.2 (10.6)	31.6 (9.6)
Female (%)	9949 (70.3%)	4432 (74%)
First recorded EDSS score (median (IQR))	2.0 (3.0)	2.5 (2)
Most recent recorded EDSS score (median (IQR))	2.5 (4.5)	3.5 (4)
Age at first recorded EDSS score (mean (SD))	42.1 (12.5)	40.8 (10.8)
Converted to secondary progressive MS during follow-up (%)	4165 (29.4%)	2127 (35%)
Duration of follow-up (median (IQR)	7.83 (5.19)	14.9 (12.9)

EDSS: expanded disability status scale; IQR: interquartile range; SD: standard deviation; MS: multiple sclerosis.

### Swedish cohort data

**Figure 2.**
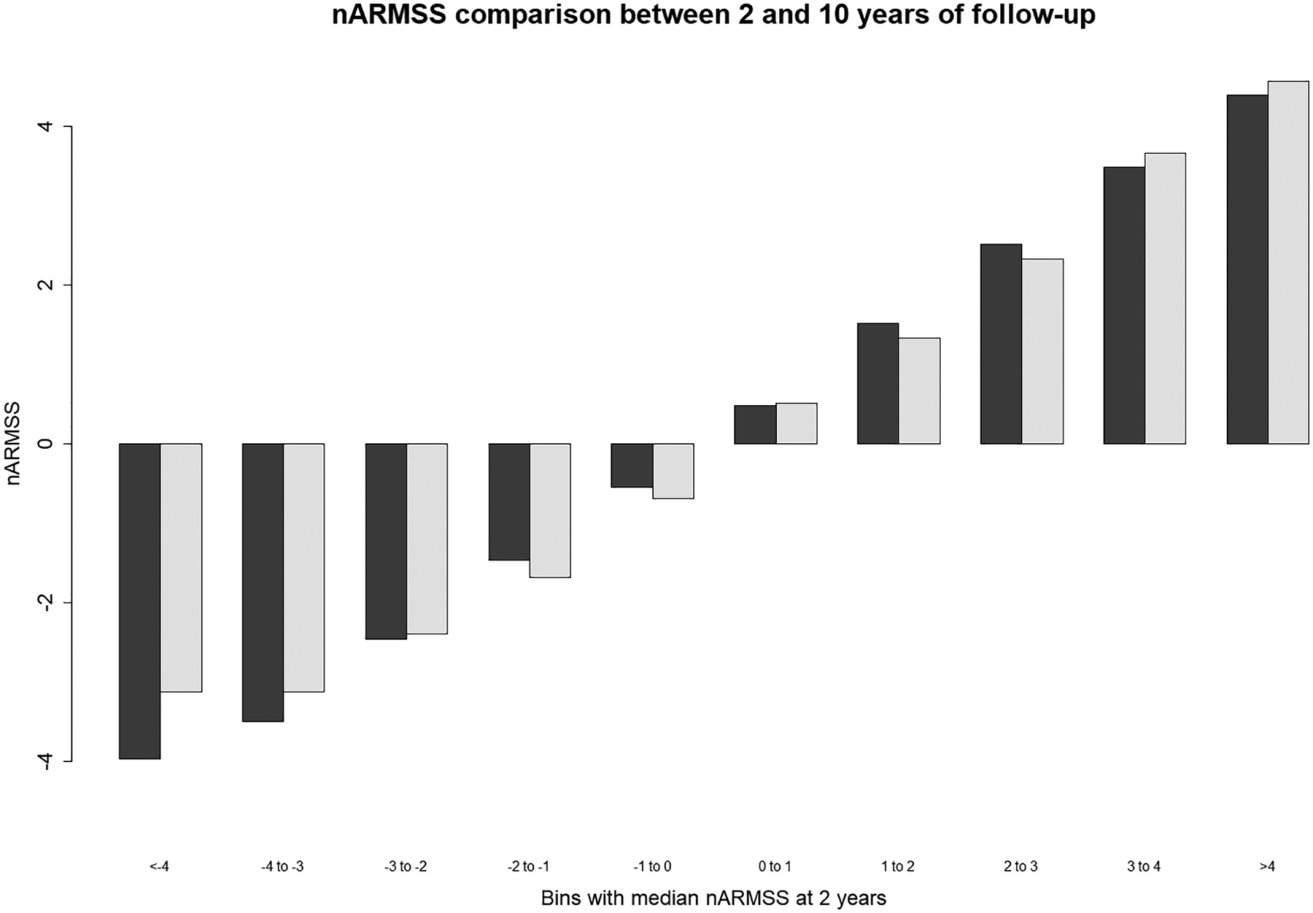
nARMSS comparison between 2 and 10 years of follow-up (*n* = 3556), without overlap.

The relationship between nARMSS from 2 to 10 years can be visualized in Figure 2. For each stratum of individuals after 2 years of follow-up, defined by nARMSS in 1-year intervals and shown in black, the corresponding values for the subsequent period are presented. The light bar gives the median values of the next 8 years of nARMSS for individuals in a given strata; that is, not using the initial 2 years of data. As can be seen in the figure, median nARMSS for the first 2 years of follow-up are very similar to the median values for the next 8 years of follow-up.

The range of nARMSS for all individuals had a median of 1.17 points over all patient visits, compared with a median range of 3.24 for the underlying ARMSS recorded. This corresponds to a ratio of the nARMSS range of 0.36 to that of ARMSS, meaning that nARMSS ranges varied less than ARMSS over the same number of measurements.

**Figure 3.**
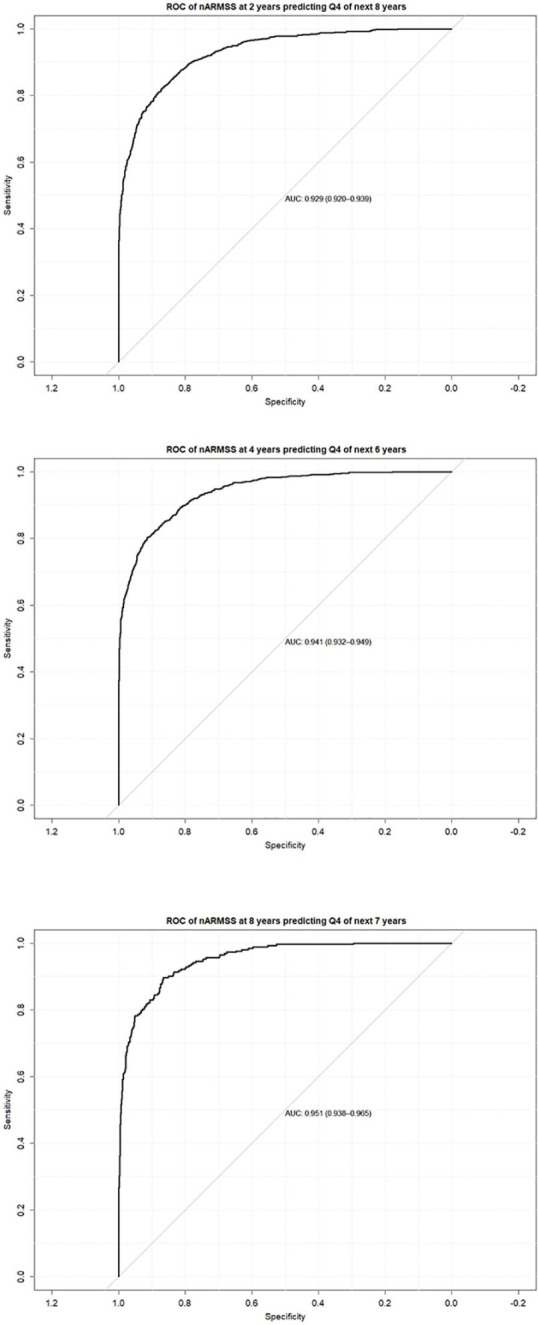
AUC for ROC of the nARMSS at (top) 2 years predicting Q4 of the nARMSS for next 8 years (*n* = 3419), (middle) 4 years predicting Q4 of the nARMSS for next 6 years and (bottom) 8 years predicting Q4 of nARMSS for next 7 years (*n* = 3489).

Moving to the individual level, the ability of the nARMSS at 2 and 4 years of follow-up to predict the most severe patients in the fourth quartile of the nARMSS at 10 years are shown in Figure 3. The AUC-ROC values were 0.929 (95% CI = 0.920–0.939, *n* = 3419) for the nARMSS at 2 years predicting the next 8 years and 0.941 (95% CI = 0.932–0.949, *n* = 3489) for the nARMSS at 4 years predicting the next 6 years.

A determination of AUC-ROC stability over longer time periods in Figure 3 using the ROC of nARMSS at 8 years predicting Q4 of the nARMSS over the next 7 years resulted in an AUC of 0.951 (95% CI = 0.938–0.965, *n* = 1006).

The ability of nARMSS at 2 and 4 years to determine whether SPMS had been reached after 10 years of follow-up is shown in Supplemental Figure 1. The nARMSS at 2 years had a 0.662 (95% CI = 0.639–0.686, *n* = 877) AUC-ROC at 10 years, while the nARMSS at 4 years had a 0.663 (95% CI = 0.638–0.687, *n* = 772) AUC-ROC. These accuracies were surpassed by other predictors, with the EDSS at 2 years having an AUC-ROC of 0.754 (95% CI = 0.734–0.775, *n* = 2646) and the EDSS at 4 years having an AUC-ROC of 0.763 (95% CI = 0.741–0.784, *n* = 2540).

Complete data on the AUC-ROC for each test made in both cohorts are given in Supplemental Table 1. Supplemental Figure 1 illustrates all additional AUC-ROC plots using both Q4 of nARMSS and SP at 10 years of follow-up.

### Validation in the Canadian cohort

Similar AUC-ROC curves were calculated in the Canadian cohort for nARMSS at 2 and 4 years to predict the most severe quartile after the subsequent years to 10 years of follow-up. These values were very similar to those of the Swedish cohort, with an AUC-ROC of 0.901 (95% CI = 0.877–0.924 for 2 years to predict next 8 years, *n* = 948) and 0.908 (95% CI = 0.886–0.929 for 4 years to classify the next 6 years, *n* = 904). All other variables used to predict the most severe nARMSS quartile showed slight reductions from the AUC-ROC values obtained in the Swedish cohort (Supplemental Table 1).

When predicting SPMS in the Canadian cohort, all variables showed slight increases compared with the Swedish cohort with the AUC-ROC obtained by nARMSS at 2 years being 0.732 (95% CI = 0.701–0.764) and at 4 years being 0.744 (95% CI = 0.712–0.775). The largest value of AUC-ROC in the Canadian cohort using SP status at 10 years was obtained with EDSS at 4 years (0.818, 95% CI = 0.790–0.845).

### Comparison to other measures of disease severity

MS cases in the most severe nARMSS quartile had substantially lower first SDMT scores (mean: 42.7, *n* = 1341) when compared to the lowest nARMSS quartile (mean: 53.3, *n* = 2130). MSIS-29 physical and psychological scores were consistently lower in nARMSS Q4 (mean physical score of 2.84, mean psychological score of 2.64, *n* = 1436) than those in nARMSS Q1 (mean physical score of 1.57, mean psychological score of 1.95, *n* = 2265). Similar trends in lower SDMT scores in the most severe quartile compared to the least severe quartile were observed with EDSS (Q4 = 38.7, *n* = 813 compared to Q1 = 54.7, *n* = 2577) and MSSS (Q4 = 41.6, *n* = 1196 compared to Q1 = 53.6, *n* = 2261).

A sub-analysis of nARMSS for those with and without missing SDMT indicated that mean nARMSS was significantly different for all quartiles. Q4 showed the largest increase in nARMSS between non-missing and missing data (quartile: available, missing, *p* value – Q1: −1.43, −1.68, *p* < 0.001, Q2: −0.33, −0.34, *p* = 0.001, Q3: −0.004, 0.004, *p* = 0.01, Q4: 0.65, 1.17, *p* < 0.001). This indicates that more severe disability in Q4 is likely under-reported and that interquartile differences might increase with more complete data. This tendency towards less favourable outcomes with increasing quartiles of nARMSS is presented in Table 2.

**Table 2.** Comparison of nARMSS, EDSS and MSSS by quartiles with the first recorded SDMT and MSIS-29 scores.

Quartiles of last nARMSS					
	Q1 (*n* = 3540)	Q2 (*n* = 3540)	Q3 (*n* = 3540)	Q4 (*n* = 3540)	All (*n* = 14,160)
nARMSS (mean (SD))	–3.89 (0.492)	–2.12 (0.537)	–0.0116 (0.714)	3.03 (1.10)	–0.76 (2.61)
Average of EDSS score measurements during follow-up (SD)	0.905 (0.796)	2.03 (1.05)	3.41 (1.47)	6.06 (1.76)	3.10 (2.33)
First SDMT score					
Mean (SD)	53.4 (11.4)	51.3 (12.2)	48.4 (13.0)	42.7 (14.2)	49.6 (13.1)
Missing, *n* (%)	1410 (39.8%)	1200 (33.9%)	1406 (39.7%)	2199 (62.1%)	6215 (43.9%)
MSIS-29					
Physical score (mean (SD))	1.45 (0.585)	1.77 (0.746)	2.22 (0.864)	2.88 (0.951)	1.99 (0.922)
Psychological score (mean (SD))	1.89 (0.825)	2.24 (0.913)	2.51 (0.964)	2.71 (1.01)	2.30 (0.967)
Missing, *n* (%)	1275 (36.0%)	1075 (30.4%)	1311 (37.0%)	2104 (59.4%)	5765 (40.7%)
Quartiles of average EDSS					
	Q1 (*n* = 3540)	Q2 (*n* = 3540)	Q3 (*n* = 3540)	Q4 (*n* = 3540)	All (*n* = 14160)
nARMSS (mean (SD))	–3.50 (0.944)	–1.79 (1.47)	–0.277 (1.74)	2.57 (1.67)	–0.748 (2.68)
Average of EDSS score measurements during follow-up (SD)	0.623 (0.415)	1.83 (0.320)	3.38 (0.662)	6.57 (1.10)	3.10 (2.33)
First SDMT score					
Mean (SD)	54.7 (11.3)	51.5 (11.6)	45.3 (12.7)	38.7 (14.1)	49.6 (13.1)
Missing, *n* (%)	963 (27.2%)	1007 (28.4%)	1518 (42.9%)	2727 (77.0%)	6215 (43.9%)
MSIS-29					
Physical score (mean (SD))	1.39 (0.528)	1.81 (0.726)	2.44 (0.843)	3.20 (0.823)	1.99 (0.922)
Psychological score (mean (SD))	1.93 (0.852)	2.30 (0.928)	2.58 (0.978)	2.69 (0.986)	2.30 (0.967)
Missing, *n* (%)	863 (24.4%)	901 (25.5%)	1378 (38.9%)	2623 (74.1%)	5765 (40.7%)
Quartiles of average MSSS					
	Q1 (*n* = 3540)	Q2 (*n* = 3540)	Q3 (*n* = 3540)	Q4 (*n* = 3540)	All (*n* = 14,160)
nARMSS (mean (SD))	–3.52 (0.940)	–1.87 (1.35)	–0.129 (1.67)	2.53 (1.80)	–0.748 (2.68)
Average of EDSS score measurements during follow-up (SD)	0.864 (0.760)	2.01 (1.00)	3.42 (1.44)	6.11 (1.68)	3.10 (2.33)
First SDMT score					
Mean (SD)	53.6 (11.7)	51.4 (11.8)	47.9 (13.0)	41.6 (14.0)	49.6 (13.1)
Missing, *n* (%)	1279 (36.1%)	1188 (33.6%)	1404 (39.7%)	2344 (66.2%)	6215 (43.9%)
MSIS-29					
Physical score (mean (SD))	1.44 (0.584)	1.76 (0.730)	2.26 (0.872)	2.95 (0.903)	1.99 (0.922)
Psychological score (mean (SD))	1.92 (0.851)	2.21 (0.910)	2.53 (0.963)	2.74 (0.985)	2.30 (0.967)
Missing, *n* (%)	1173 (33.1%)	1072 (30.3%)	1309 (37.0%)	2211 (62.5%)	5765 (40.7%)

ARMSS: age-related MS severity score; EDSS: expanded disability status scale; SDMT: Symbol Digit Modalities Test; MSIS-29: MS impact scale-29; SD: standard deviation.

## Discussion

The AUC-ROCs show that the nARMSS has a strong capacity to predict future disability, even when only 2 years of follow-up is available. This allows the nARMSS to be used when categorizing individuals in severity studies with an improvement in accuracy when compared to the use of cross-sectional metrics such as the first or the last available EDSS/ARMSS or MSSS. A potential use of the nARMSS is as an outcome measure in studies such as genome wide association studies (GWAS) for disease severity and progression. In this setting, where severity is not stable and therefore noisy, isolating the signal using the nARMSS may allow for more accurate and reproducible results. Patients can also be included in research studies regardless of their age at measurement and follow-up time, without affecting the results due to large fluctuations in these factors.

When comparing the ranges for the ARMSS and nARMSS for patients over the entire course of their follow-up, the nARMSS in effect compresses the variability of values. The median nARMSS variation is approximately a third of that of the variation in ARMSS measurements, denoting close to a two-third reduction in the instability of serial EDSS/ARMSS measurements. It is precisely this reduction in variation, despite the similar scales, which gives the nARMSS increased utility as an overall marker of disease progression even when determined early in follow-up.

The reasons for using the ARMSS to construct such a metric, instead of the MSSS are (1) the potential increased size of the available patient pool, since recorded patient information may lack onset date but not age and (2) the elimination of the risk of systematic bias due to retrospectively assigning the date of onset. While the nARMSS has some power to predict SPMS after 10 years, EDSS scores alone have increased AUC-ROCs, likely due to the fact that individuals with high EDSS after 2 or 4 years of follow-up are more likely to convert to SP within 10 years. Since we have removed individuals with SP at the measurement point, these patients who remained and had high EDSS are closer to phenotype conversion. Other more accurate methods of predicting SPMS exist, which exceed the accuracy demonstrated here.^14^

Similarly, in a clinical setting, the nARMSS may be useful as an additional data point for neurologists to use in combination with other factors and might be used to determine if a patient is likely to have a milder or more severe disease course early in treatment. Given data showing the importance of early treatment, this could be useful in clinical practice when making treatment decisions for early diagnosed patients.^15^ However, since there are always exceptions to strong trends, clinical application should be undertaken with caution.

The main limitation of the nARMSS is that it is constructed from EDSS measurements and thus biased towards mobility, especially at higher scores (>5) which consist solely of physical factors. Cognitive disability, for example, is less well represented. Additional outcomes might better represent all aspects of the disease. For example, income and sickness-absence data are available in Sweden, and both are correlated with cognitive decline.^16^ However, comparisons between the nARMSS quartiles and first SDMT scores show negative correlation (Table 2), which as expected implies that cognitive decline is associated with the nARMSS as both indicate disability and SDMT may have a predictive role on motor disability. Similarly, both MSIS-29 physical and psychological scores showed correlation with the nARMSS, providing further confirmation of utility beyond only physical disability. Nevertheless, composite scores with SDMT, MSIS-29 and other metrics might lead to greater accuracies. However, it should be noted that relying on EDSS has the benefit of including nearly all patients due to the large data availability of such a metric.

While the correlation between the nARMSS early in follow-up and after 10+ years of follow-up is high, it should be noted from Figure 1 that the nARMSS does vary over a patient’s disease course, ultimately reaching a nearly steady-state level. Therefore, it can be inferred that the metric becomes more accurate with more follow-up time, likely due to more complete information. Individuals enrolled into severity studies should use the most recent clinical measurement available to calculate nARMSS for greatest accuracy.

This metric could aid in the search for factors which are correlated with MS disease severity, such as genetic markers which are associated with increased disease severity. Furthermore, early identification of the potential future severity of an individual’s disease could inform the most appropriate treatment option(s) for that patient. Finally, any alterations in disease progression could be more accurately captured, so that interventions and factors which improve disease course could be identified.

## Supplemental Material

sj-pdf-1-msj-10.1177_1352458520988632 – Supplemental material for A multiple sclerosis disease progression measure based on cumulative disability Click here for additional data file. Supplemental material, sj-pdf-1-msj-10.1177_1352458520988632 for A multiple sclerosis disease progression measure based on cumulative disability by Ali Manouchehrinia, Elaine Kingwell, Feng Zhu, Helen Tremlett, Jan Hillert and Ryan Ramanujam in Multiple Sclerosis Journal

sj-pdf-2-msj-10.1177_1352458520988632 – Supplemental material for A multiple sclerosis disease progression measure based on cumulative disability Click here for additional data file. Supplemental material, sj-pdf-2-msj-10.1177_1352458520988632 for A multiple sclerosis disease progression measure based on cumulative disability by Ali Manouchehrinia, Elaine Kingwell, Feng Zhu, Helen Tremlett, Jan Hillert and Ryan Ramanujam in Multiple Sclerosis Journal

## References

[1] Kurtzke JF. Rating neurologic impairment in multiple sclerosis: An expanded disability status scale (EDSS). Neurology 1983; 33(11): 1444–1452. 6685237 10.1212/wnl.33.11.1444

[2] Pachner AR Steiner I. The multiple sclerosis severity score (MSSS) predicts disease severity over time. J Neurol Sci 2009; 278(1–2): 66–70. 19138773 10.1016/j.jns.2008.11.020

[3] Manouchehrinia A Westerlind H Kingwell E , et al. Age related multiple sclerosis severity score: Disability ranked by age. Mult Scler J 2017; 23: 1938–1946. 10.1177/1352458517690618 PMC5700773 28155580

[4] Kosa P Ghazali D Tanigawa M , et al. Development of a sensitive outcome for economical drug screening for progressive multiple sclerosis treatment. Front Neurol 2016; 7: 131–114. 10.3389/fneur.2016.00131 PMC4983704 27574516

[5] Weideman AM Barbour C Tapia-Maltos MA , et al. New multiple sclerosis disease severity scale predicts future accumulation of disability. Front Neurol 2017; 8: 598. 29176958 10.3389/fneur.2017.00598 PMC5686060

[6] Simonian NA McAllister A Lull J , et al. ‘Summary measure’ statistic for assessing the outcome of treatment trials in relapsing-remitting multiple sclerosis (multiple letters). J Neurol Neurosurg Psychiatry 1999; 664: 553–554. 10.1136/jnnp.66.4.553 PMC1736303 10201444

[7] Andersen O . From the Gothenburg cohort to the Swedish multiple sclerosis registry. Acta Neurol Scand Suppl 2012(195): 13–19. 10.1111/ane.12023 23278651

[8] Hillert J Stawiarz L. The Swedish MS registry – Clinical support tool and scientific resource. Acta Neurol Scand 2015; 132(199): 11–19. 26046553 10.1111/ane.12425 PMC4657484

[9] Tremlett H Paty D Devonshire V. Disability progression in multiple sclerosis is slower than previously reported. Neurology 2006; 66(2): 172–177. 16434648 10.1212/01.wnl.0000194259.90286.fe

[10] Tremlett H Yinshan Zhao Devonshire V. Natural history of secondary-progressive multiple sclerosis. Mult Scler 2008; 14(3): 314–324. 18208898 10.1177/1352458507084264

[11] Smith A. Symbol digit modalities test: manual. Los Angeles, CA: Western Psychological Services, 1982.

[12] Hobart J Lamping D Fitzpatrick R , et al. The multiple sclerosis impact scale (MSIS-29): A new patient-based outcome measure. Brain 2001; 124(Pt. 5): 962–973. 11335698 10.1093/brain/124.5.962

[13] McKay KA Manouchehrinia A Berrigan L , et al. Long-term cognitive outcomes in patients with pediatric-onset vs adult-onset multiple sclerosis. JAMA Neurol 2019; 769: 1028–1034. 10.1001/jamaneurol.2019.1546 PMC6580443 31206130

[14] Manouchehrinia A Zhu F Piani-Meier D , et al. Predicting risk of secondary progression in multiple sclerosis: A nomogram. Mult Scler 2019; 25: 1102–1112. 29911467 10.1177/1352458518783667

[15] Kavaliunas A Danylaite Karrenbauer V Gyllensten H , et al. Cognitive function is a major determinant of income among multiple sclerosis patients in Sweden acting independently from physical disability. Mult Scler J 2017; 25: 104–112. 10.1177/1352458517740212 29143553

[16] Kavaliunas A Tinghög P Friberg E , et al. Cognitive function predicts work disability among multiple sclerosis patients. Mult Scler J: Exp Transl Clin 2019; 51: 205521731882213. 10.1177/2055217318822134 PMC6350142 30729025

