# Supplementary material for: A multiple sclerosis disease progression measure based on cumulative disability
Source: Mult Scler. 2021 Jan 25;27(12):1875–83. doi: 10.1177/1352458520988632 (PMC8521354; doi:10.1177/1352458520988632)
Supplement: sj-pdf-1-msj-10.1177_1352458520988632 – Supplemental material for A multiple sclerosis disease progression measure based on cumulative disability [file sj-pdf-1-msj-10.1177_1352458520988632.pdf]

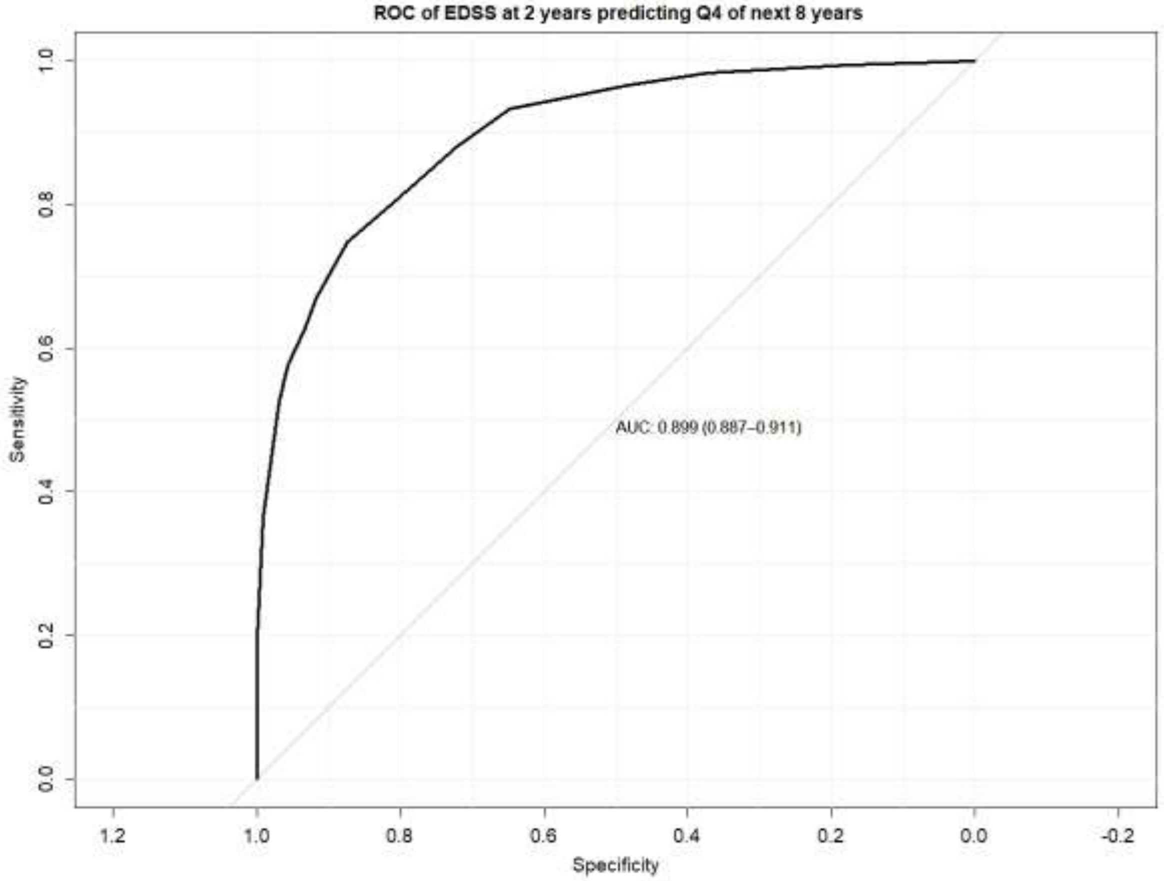
ROC of EDSS at 2 years predicting Q4 of next 8 years
Sensitivity
0.0
0.2
0.4
0.6
0.8
1.0
AUC: 0.899 (0.887–0.911)
1.2
1.0
0.8
0.6
0.4
0.2
0.0
-0.2
Specificity

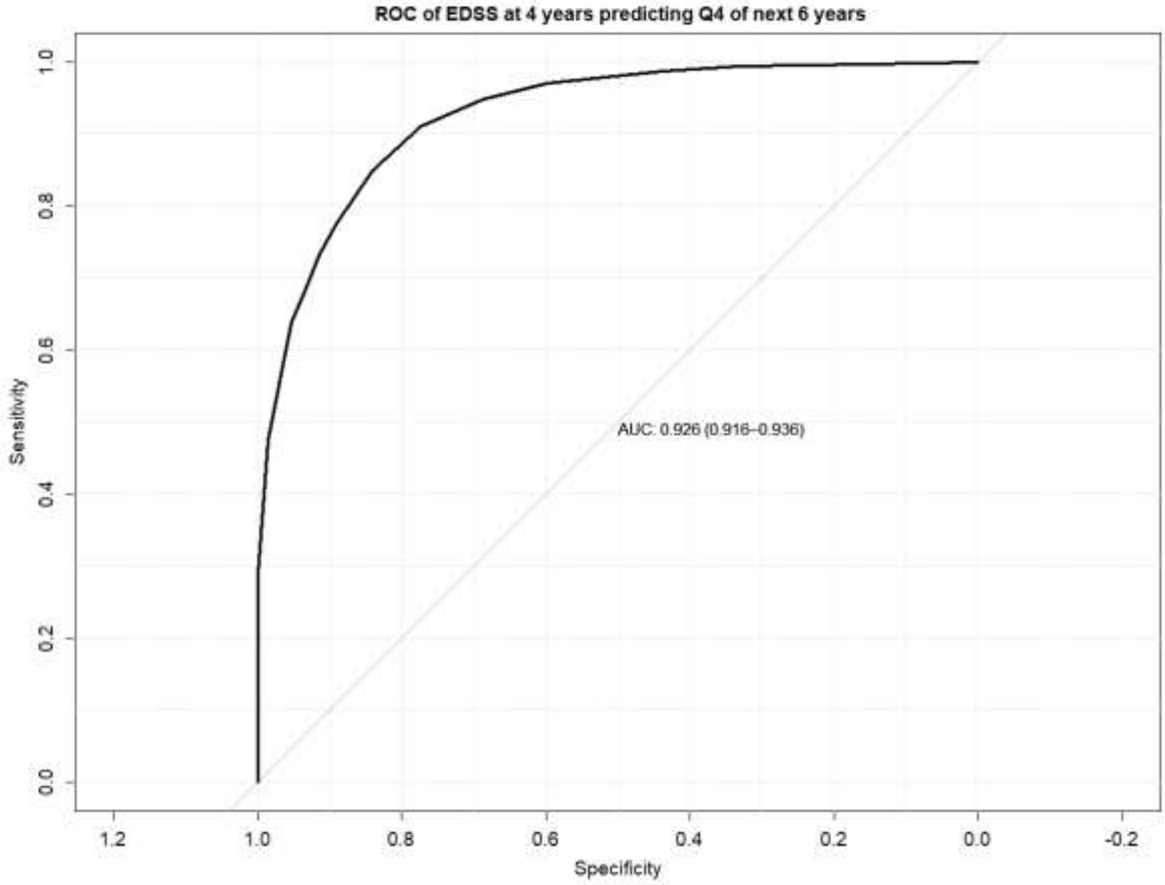
ROC of EDSS at 4 years predicting Q4 of next 6 years
Sensitivity
0.0
0.2
0.4
0.6
0.8
1.0
AUC: 0.926 (0.916–0.936)
1.2
1.0
0.8
0.6
0.4
0.2
0.0
-0.2
Specificity

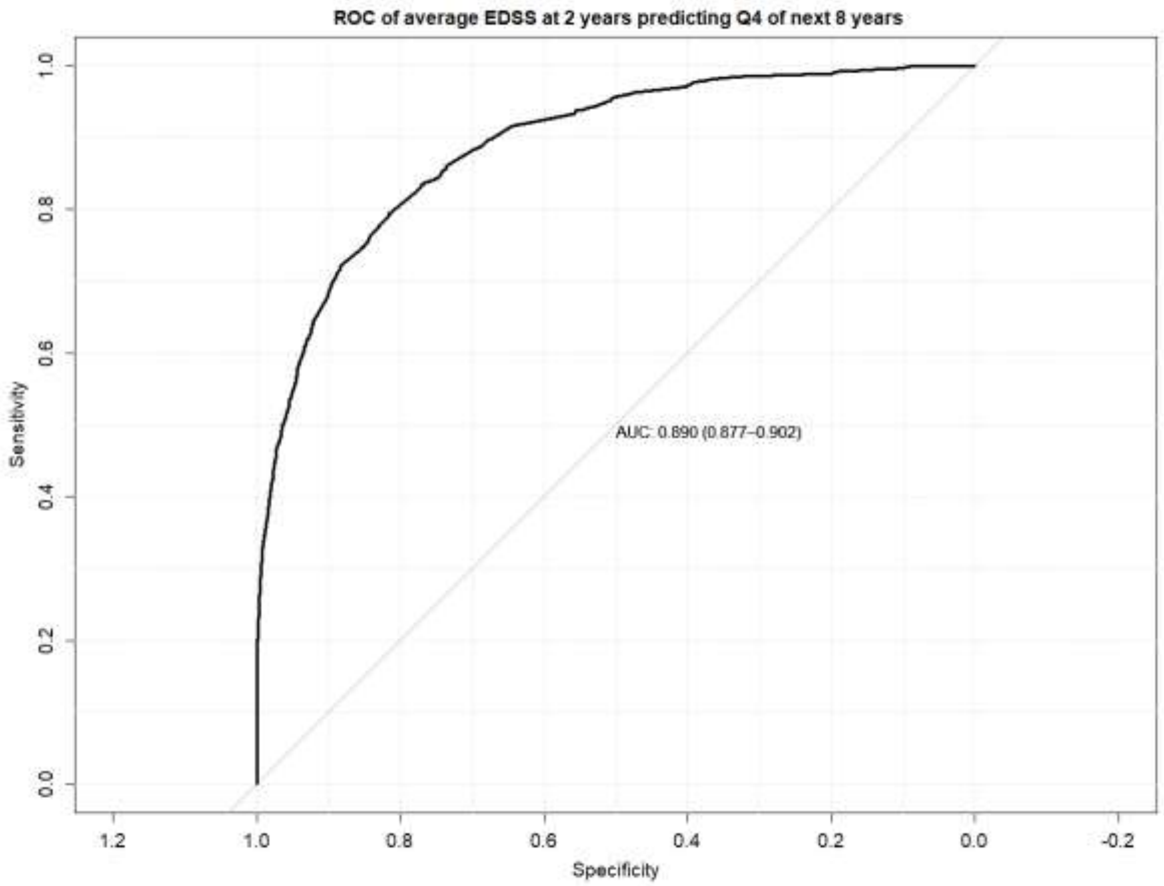
ROC of average EDSS at 2 years predicting Q4 of next 8 years
Sensitivity
Specificity
AUC: 0.890 (0.877–0.902)
1.0
0.8
0.6
0.4
0.2
0.0
1.2
1.0
0.8
0.6
0.4
0.2
0.0
-0.2

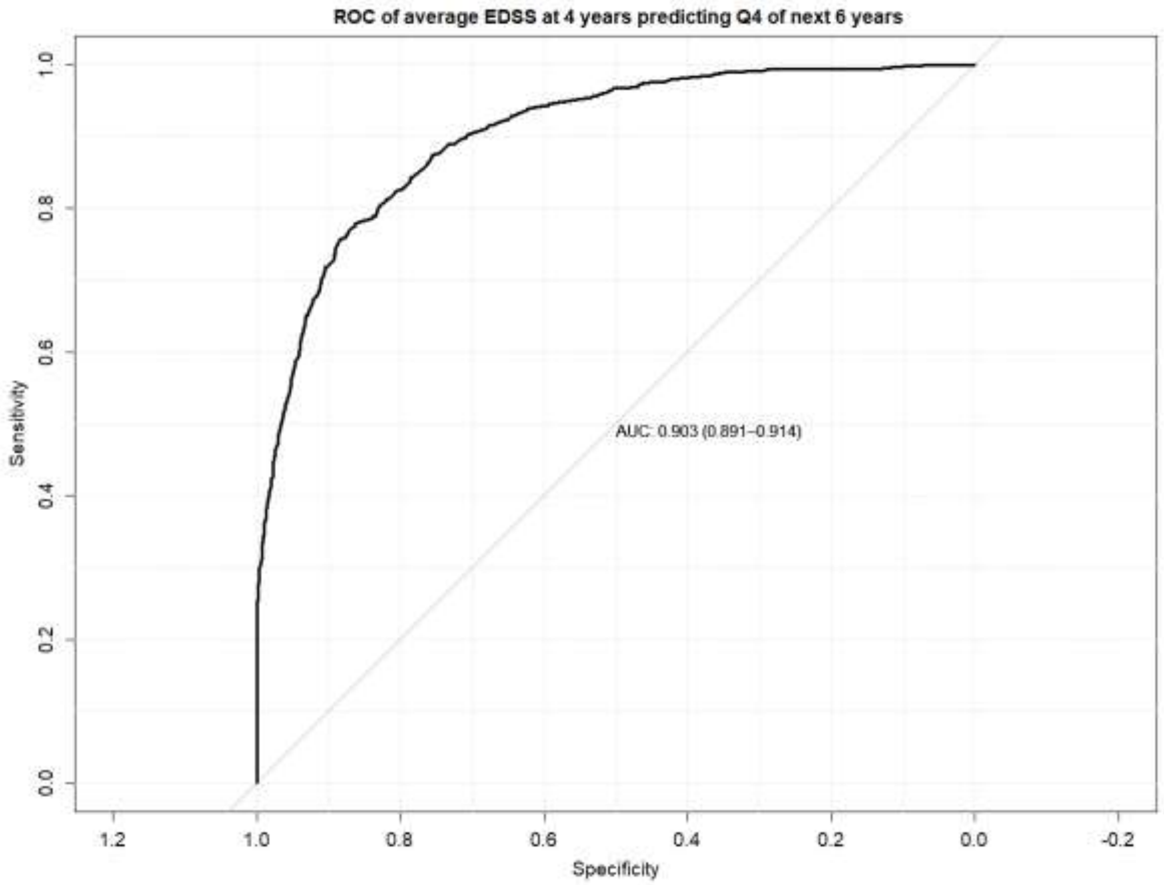
ROC of average EDSS at 4 years predicting Q4 of next 6 years
Sensitivity
Specificity
AUC: 0.903 (0.891–0.914)
1.0
0.8
0.6
0.4
0.2
0.0
1.2
1.0
0.8
0.6
0.4
0.2
0.0
-0.2

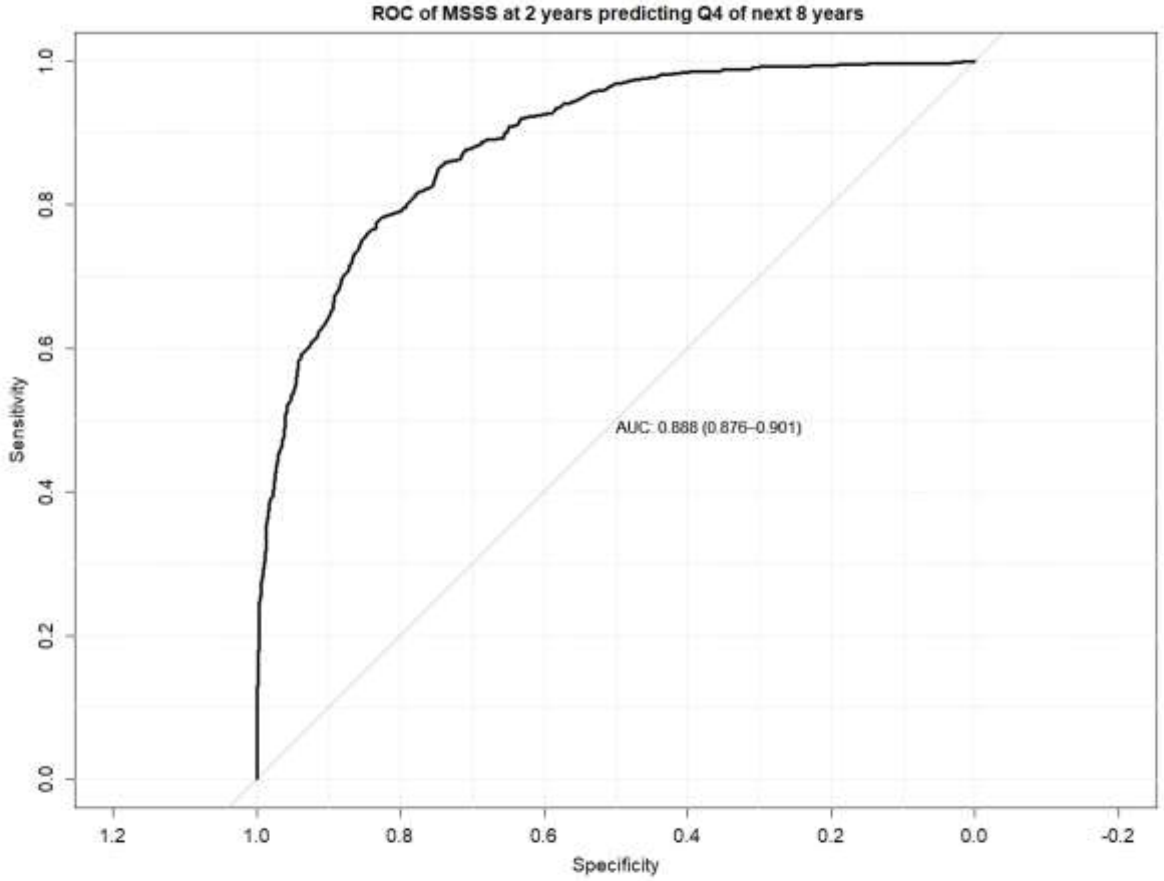

ROC of MSSS at 2 years predicting Q4 of next 8 years
AUC: 0.888 (0.876–0.901)
Sensitivity
Specificity
1.2
1.0
0.8
0.6
0.4
0.2
0.0
-0.2

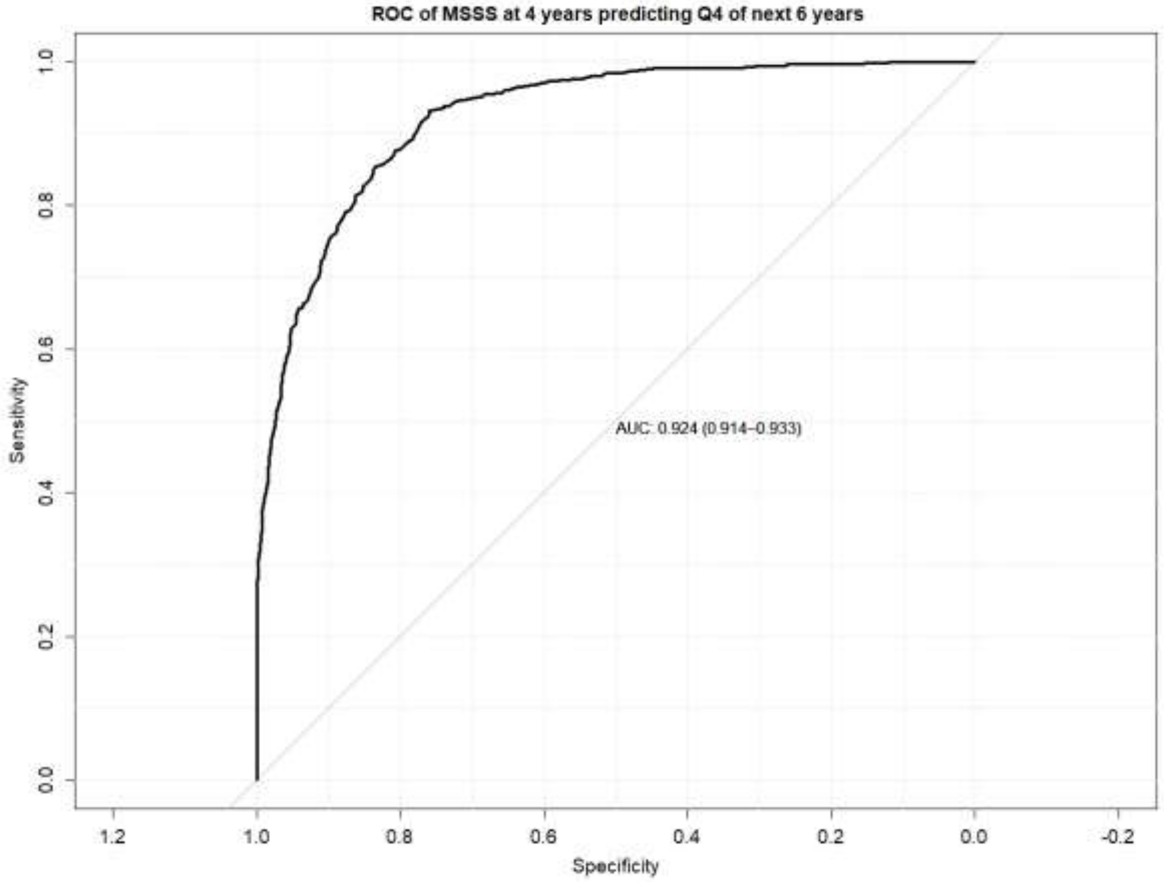

ROC of MSSS at 4 years predicting Q4 of next 6 years
AUC: 0.924 (0.914–0.933)
Sensitivity
Specificity
1.2
1.0
0.8
0.6
0.4
0.2
0.0
-0.2

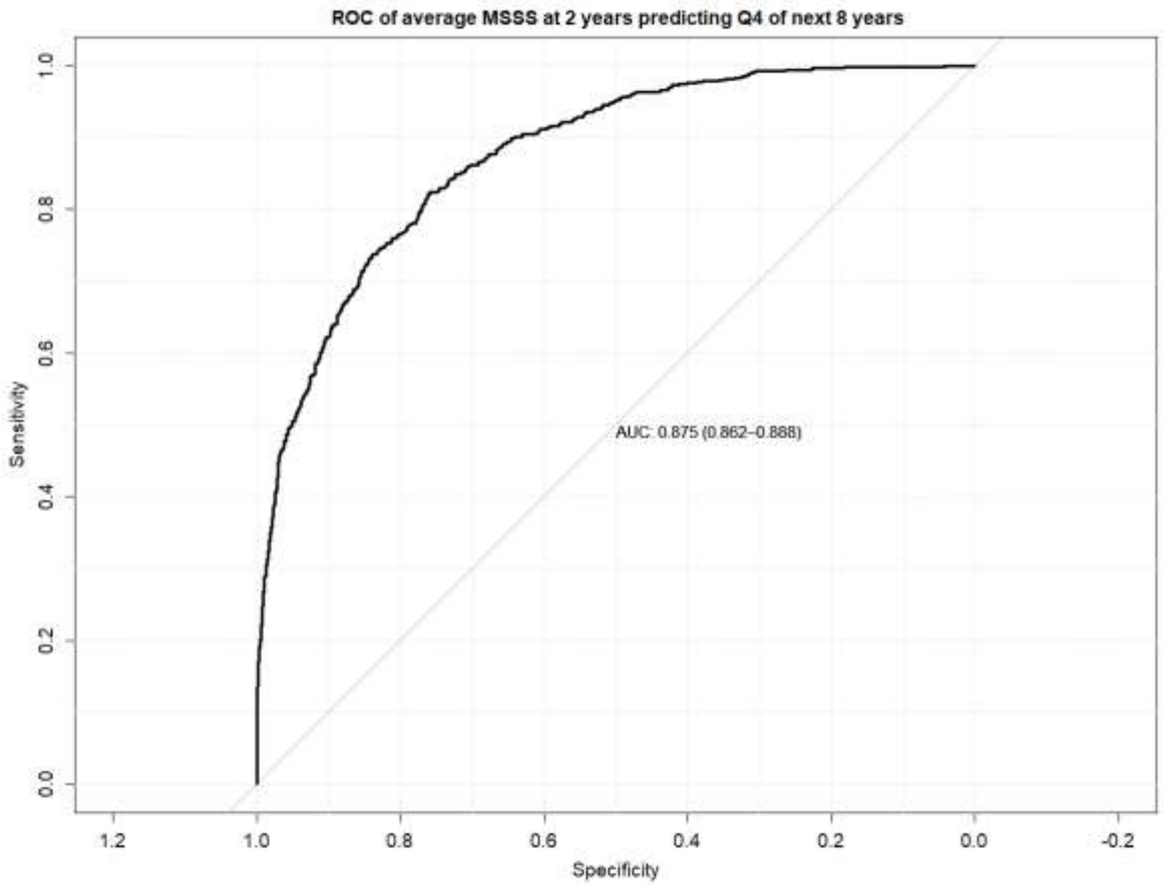
ROC of average MSSS at 2 years predicting Q4 of next 8 years
Sensitivity
Specificity
AUC: 0.875 (0.862–0.888)
1.2
1.0
0.8
0.6
0.4
0.2
0.0
-0.2

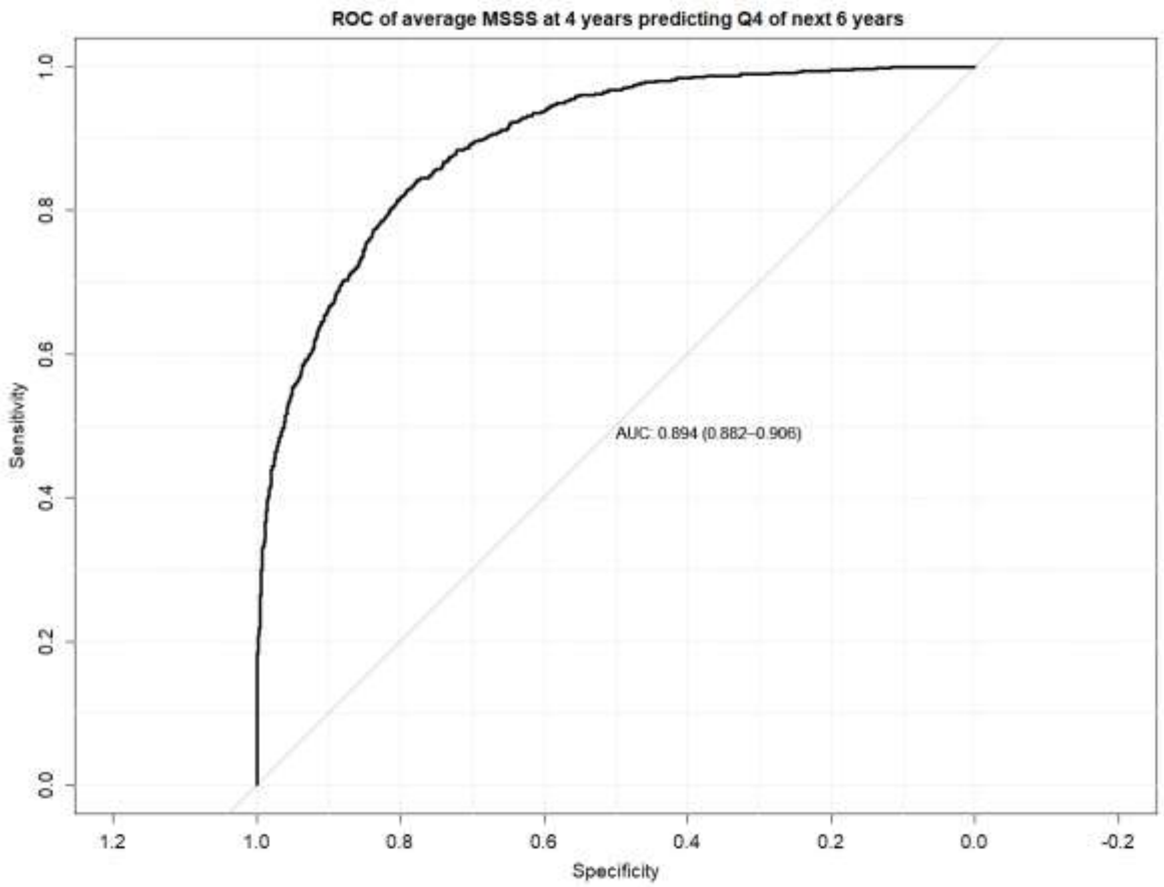
ROC of average MSSS at 4 years predicting Q4 of next 6 years
Sensitivity
Specificity
AUC: 0.894 (0.882–0.906)
1.2
1.0
0.8
0.6
0.4
0.2
0.0
-0.2

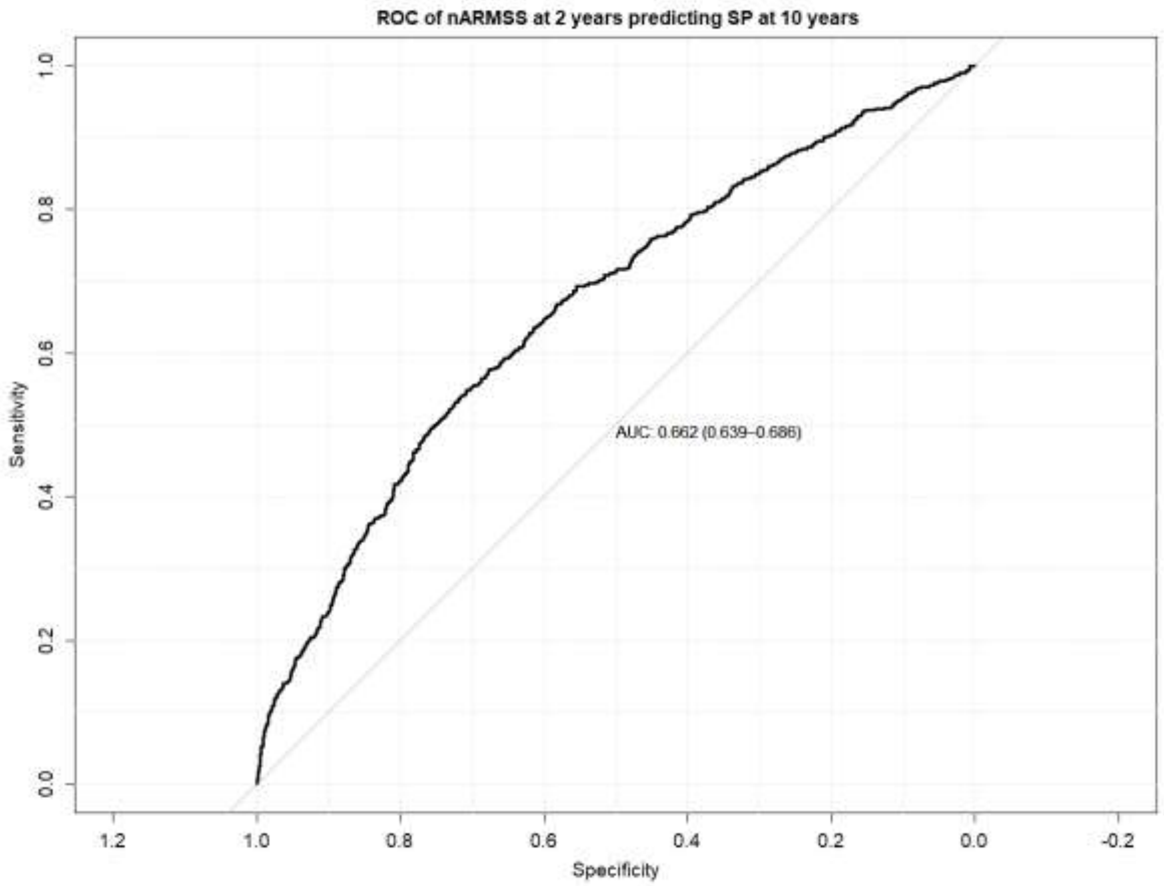

ROC of nARMSS at 2 years predicting SP at 10 years
Sensitivity
Specificity
AUC: 0.662 (0.639–0.686)
1.2
1.0
0.8
0.6
0.4
0.2
0.0
-0.2

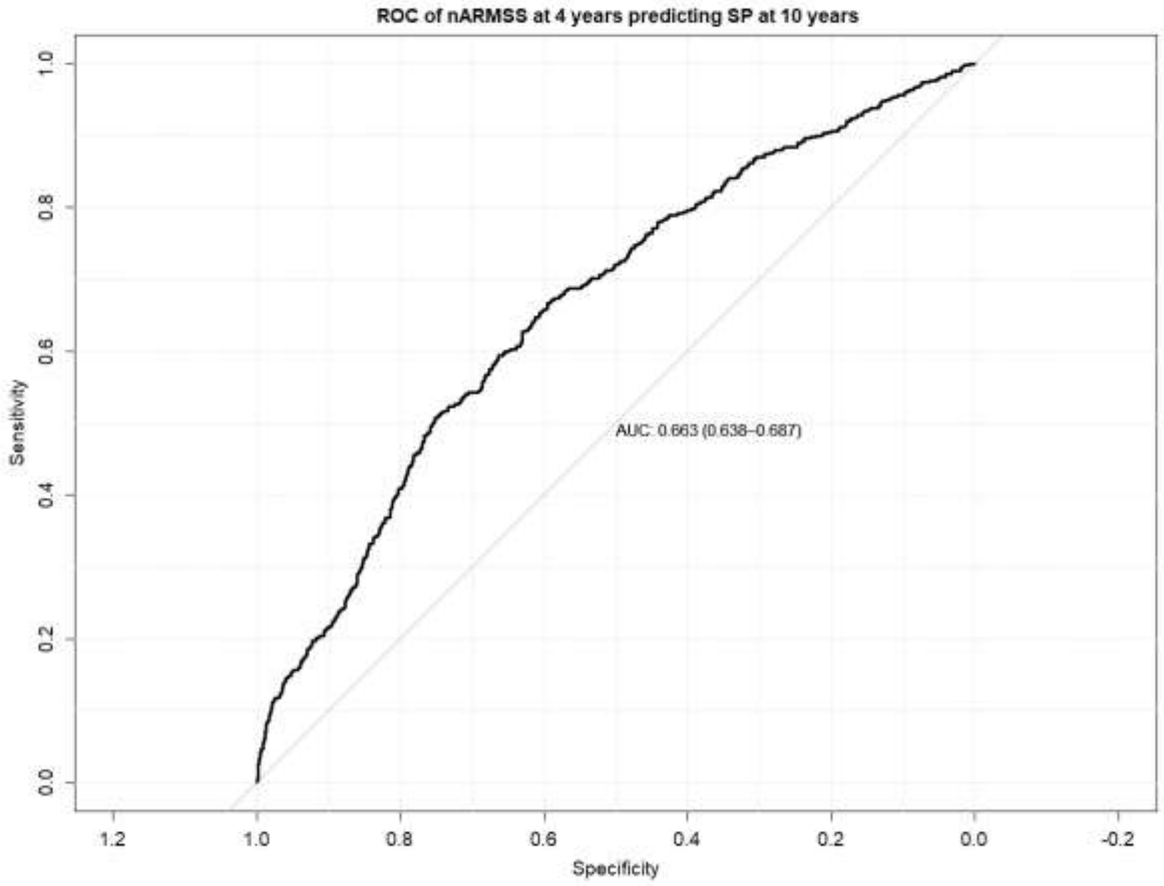

ROC of nARMSS at 4 years predicting SP at 10 years
Sensitivity
Specificity
AUC: 0.663 (0.638–0.687)
1.2
1.0
0.8
0.6
0.4
0.2
0.0
-0.2

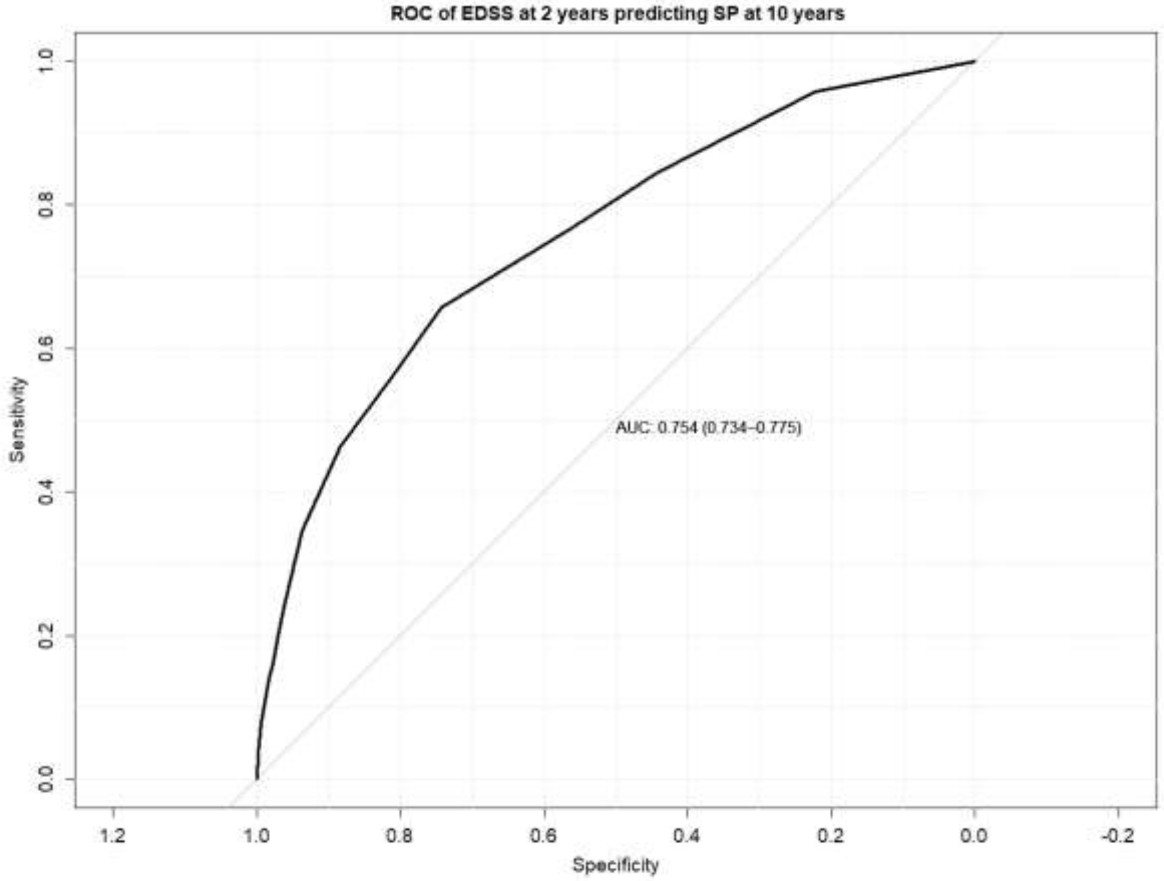
ROC of EDSS at 2 years predicting SP at 10 years
Sensitivity
Specificity
AUC: 0.754 (0.734–0.775)
1.0
0.8
0.6
0.4
0.2
0.0
1.2
-0.2

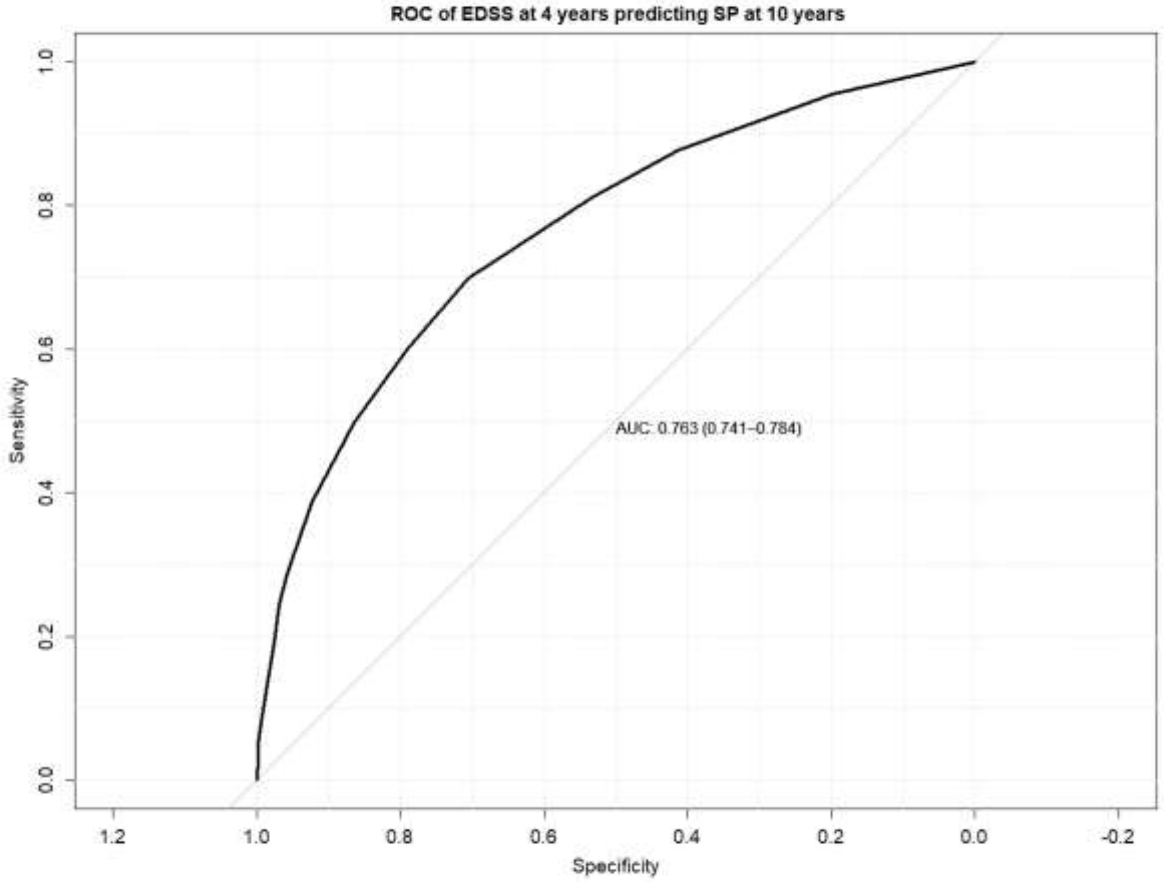
ROC of EDSS at 4 years predicting SP at 10 years
Sensitivity
Specificity
AUC: 0.763 (0.741–0.784)
1.0
0.8
0.6
0.4
0.2
0.0
1.2
-0.2

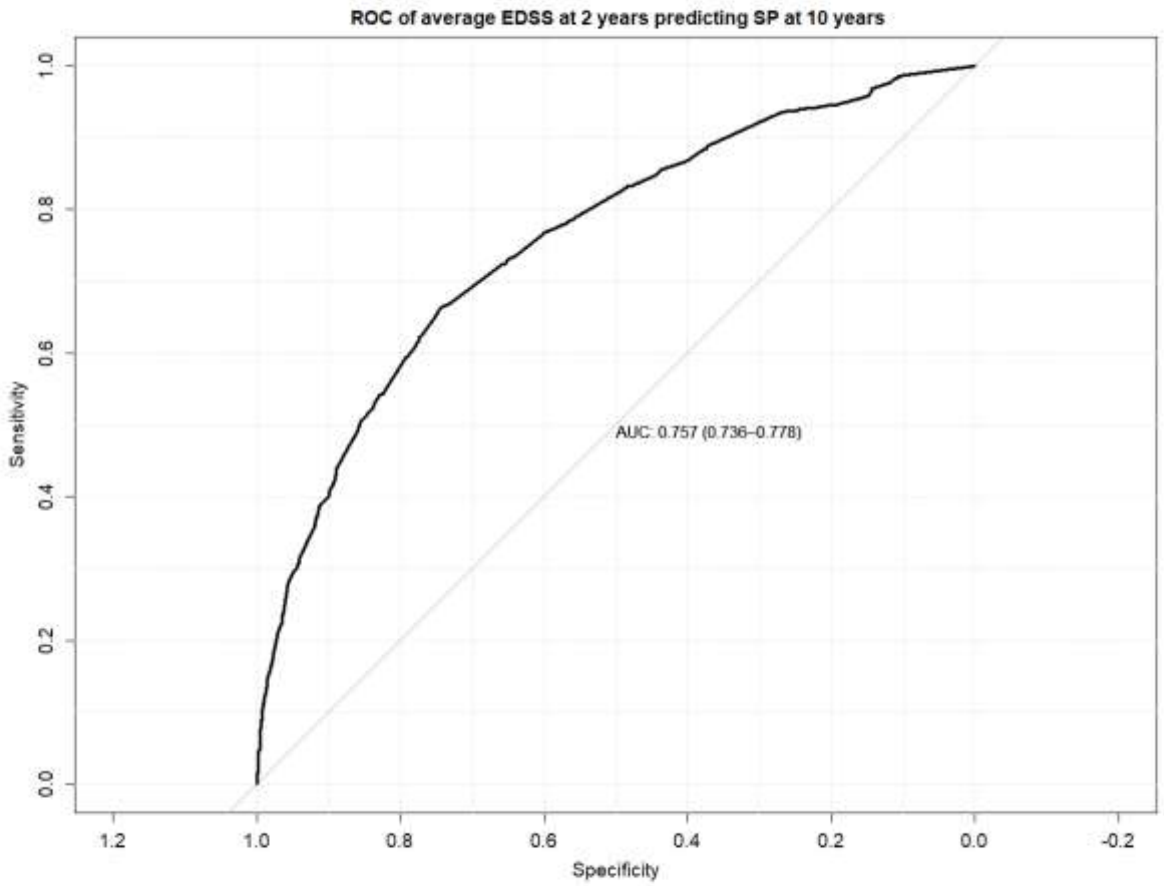
ROC of average EDSS at 2 years predicting SP at 10 years
Sensitivity
Specificity
AUC: 0.757 (0.736–0.778)
0.0
0.2
0.4
0.6
0.8
1.0
1.2
1.0
0.8
0.6
0.4
0.2
0.0
-0.2

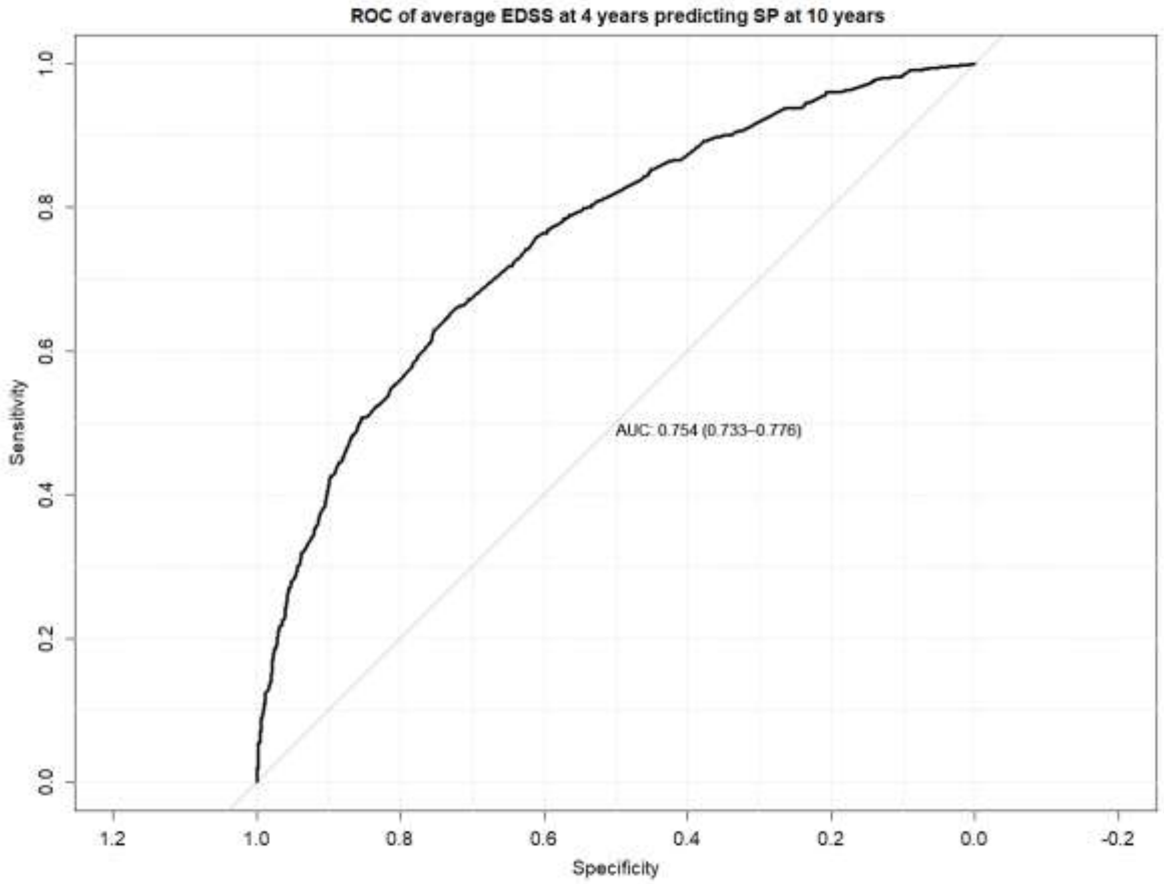
ROC of average EDSS at 4 years predicting SP at 10 years
Sensitivity
Specificity
AUC: 0.754 (0.733–0.776)
0.0
0.2
0.4
0.6
0.8
1.0
1.2
1.0
0.8
0.6
0.4
0.2
0.0
-0.2

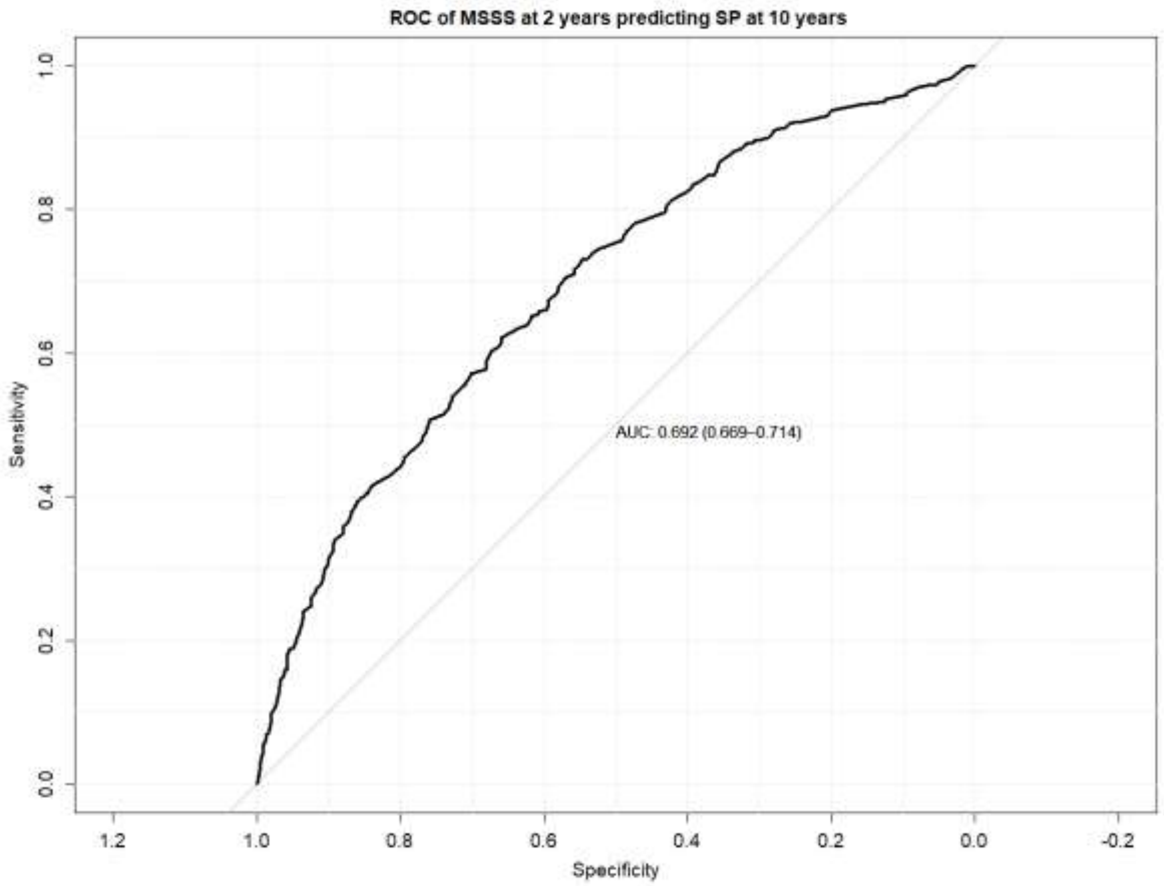
ROC of MSSS at 2 years predicting SP at 10 years
Sensitivity
Specificity
AUC: 0.692 (0.669–0.714)
1.2
1.0
0.8
0.6
0.4
0.2
0.0
-0.2

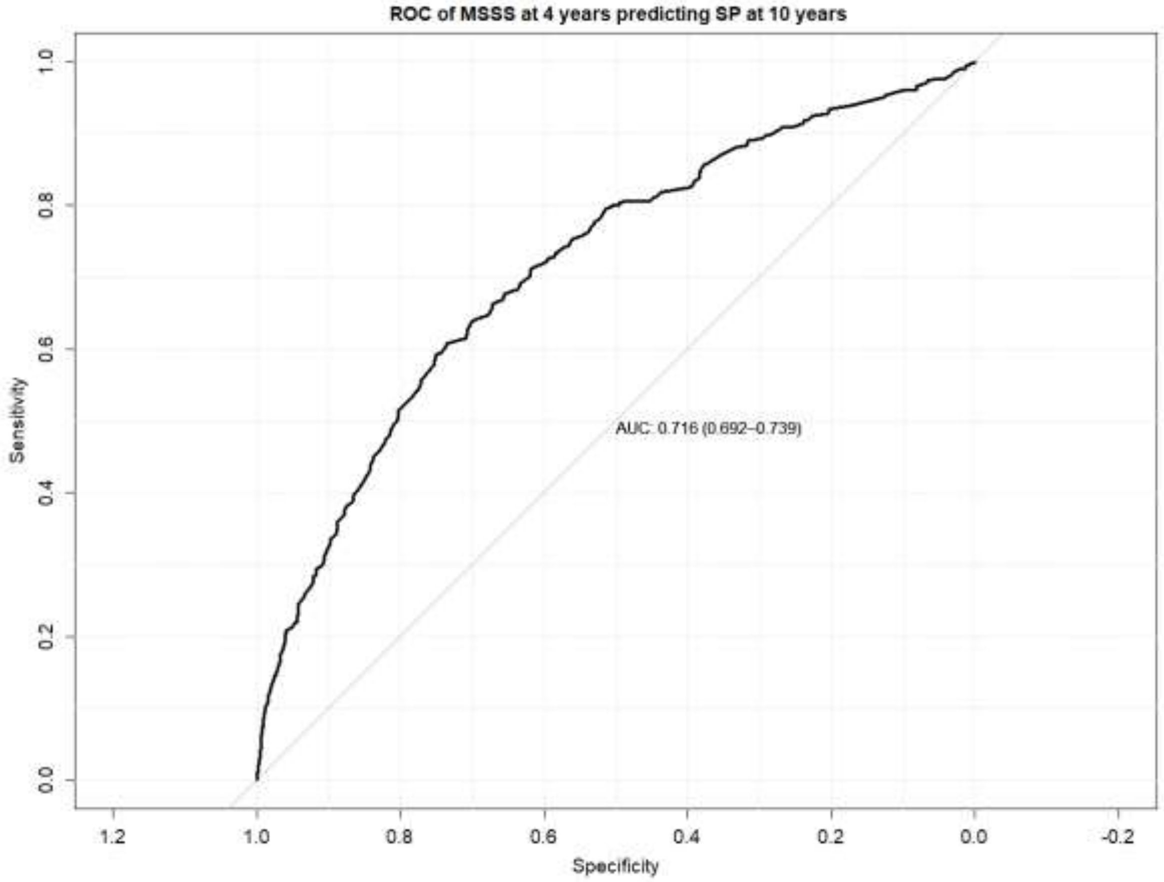
ROC of MSSS at 4 years predicting SP at 10 years
Sensitivity
Specificity
AUC: 0.716 (0.692–0.739)
1.2
1.0
0.8
0.6
0.4
0.2
0.0
-0.2

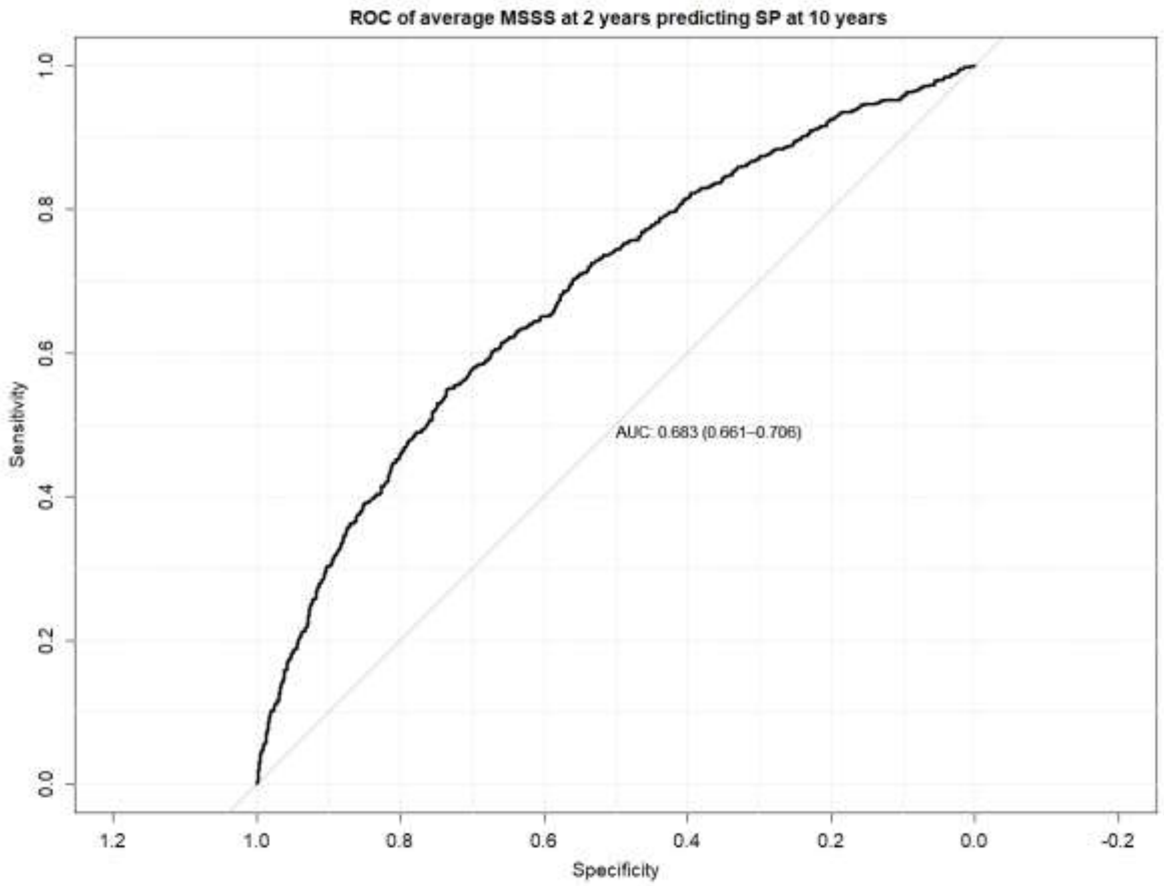
ROC of average MSSS at 2 years predicting SP at 10 years
AUC: 0.683 (0.661–0.706)
Sensitivity
Specificity
1.0
0.8
0.6
0.4
0.2
0.0
1.2
1.0
0.8
0.6
0.4
0.2
0.0
-0.2

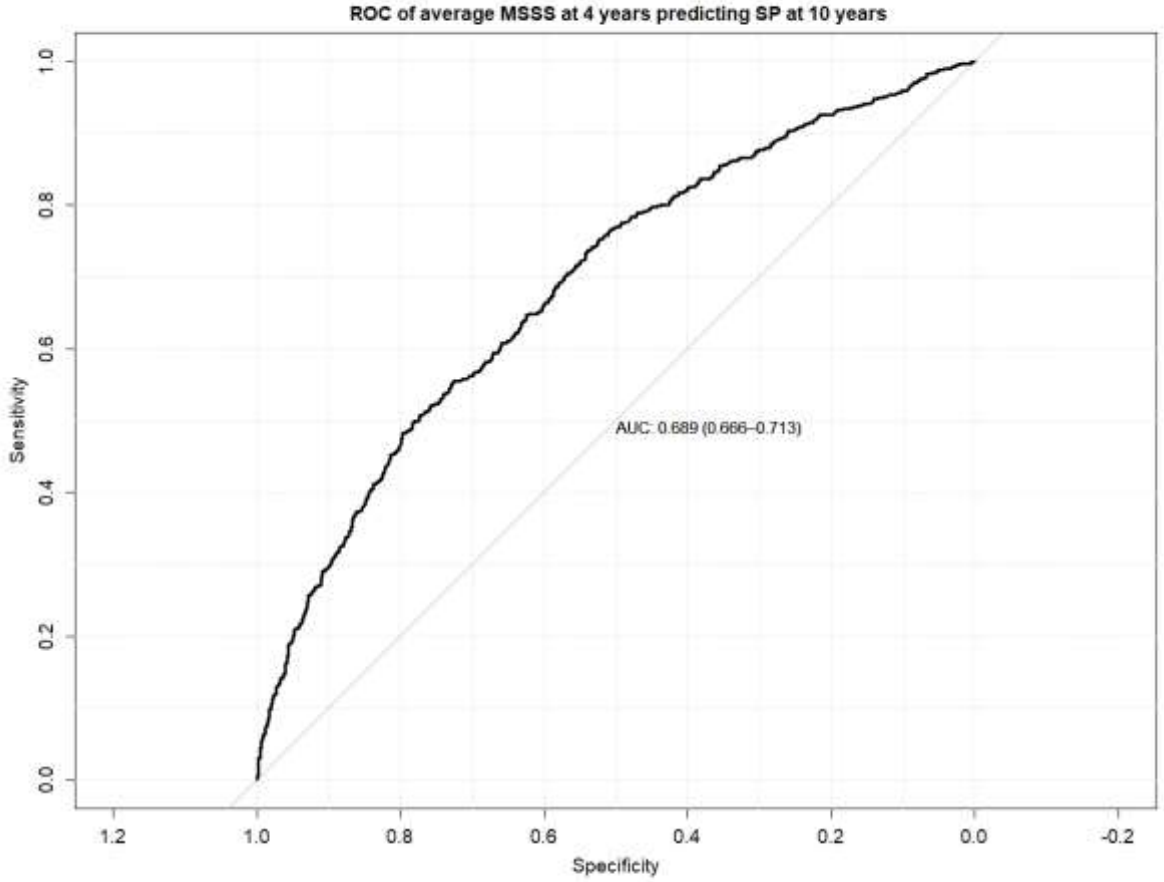
ROC of average MSSS at 4 years predicting SP at 10 years
AUC: 0.689 (0.666–0.713)
Sensitivity
Specificity
1.0
0.8
0.6
0.4
0.2
0.0
1.2
1.0
0.8
0.6
0.4
0.2
0.0
-0.2

Supplemental Figure 1. AUC-ROC plots for all tested variables and outcomes not in the main text for the Swedish cohort. The first set of plots are based on the worst quartile of nARMSS after 10 years of follow-up, without overlap. The next set of plots show prediction of SPMS after 10 years of follow-up using the same variable predictors.
