# Supplementary material for: A multiple sclerosis disease progression measure based on cumulative disability
Source: Mult Scler. 2021 Jan 25;27(12):1875–83. doi: 10.1177/1352458520988632 (PMC8521354; doi:10.1177/1352458520988632)
Supplement: sj-pdf-2-msj-10.1177_1352458520988632 – Supplemental material for A multiple sclerosis disease progression measure based on cumulative disability [file sj-pdf-2-msj-10.1177_1352458520988632.pdf]

Supplemental Table 1 - Comparison of AUC of the ROC using various variables to predict nARMSS and disease phenotype at 10 years

**Outcome - Q4 nARMSS***	**Swedish cohort**	**Canadian cohort**
nARMSS 2 years	0.929 (0.920-0.939)	0.901 (0.877-0.924)
nARMSS 4 years	0.941 (0.932-0.949)	0.908 (0.886-0.929)
nARMSS 8 years**	0.951 (0.938-0.965)	0.909 (0.873-0.945)
EDSS 2 years	0.899 (0.887-0.911)	0.875 (0.851-0.900)
EDSS 4 years	0.926 (0.916-0.936)	0.909 (0.889-0.929)
Average EDSS 2 years	0.890 (0.877-0.902)	0.856 (0.829-0.884)
Average EDSS 4 years	0.903 (0.891-0.914)	0.876 (0.851-0.900)
MSSS 2 years	0.888 (0.876-0.901)	0.869 (0.843-0.894)
MSSS 4 years	0.924 (0.914-0.933)	0.910 (0.890-0.930)
Average MSSS 2 years	0.875 (0.862-0.888)	0.845 (0.817-0.873)
Average MSSS 4 years	0.894 (0.882-0.906)	0.865 (0.840-0.891)
**Outcome – RR/SP status*** **	**Swedish cohort**	**Canadian cohort**
nARMSS 2 years	0.662 (0.639-0.686)	0.732 (0.701-0.764)
nARMSS 4 years	0.663 (0.638-0.687)	0.744 (0.712-0.775)
EDSS 2 years	0.754 (0.734-0.775)	0.799 (0.771-0.827)
EDSS 4 years	0.763 (0.741-0.784)	0.818 (0.790-0.845)
Average EDSS 2 years	0.757 (0.736-0.778)	0.797 (0.769-0.825)
Average EDSS 4 years	0.754 (0.733-0.776)	0.810 (0.783-0.838)
MSSS 2 years	0.692 (0.669-0.714)	0.728 (0.696-0.760)
MSSS 4 years	0.716 (0.692-0.739)	0.753 (0.722-0.784)
Average MSSS 2 years	0.683 (0.661-0.706)	0.710 (0.677-0.743)
Average MSSS 4 years	0.689 (0.666-0.713)	0.722 (0.689-0.755)

*Outcome was most severe quartile (Q4) nARMSS for period from 2 and 4 years to 10, respectively, without overlap.

** For only this test, outcome was was most severe quartile nARMSS for the period from 8-15 years of follow-up.

** Outcome was phenotype at 10 years of follow-up.
